# Epidermal necrolysis French national diagnosis and care protocol (PNDS; *protocole national de diagnostic et de soins*)

**DOI:** 10.1186/s13023-018-0793-7

**Published:** 2018-04-10

**Authors:** Saskia Ingen-Housz-Oro, Tu-Anh Duong, Benoit Bensaid, Nathalia Bellon, Nicolas de Prost, Dévy Lu, Bénédicte Lebrun-Vignes, Julie Gueudry, Emilie Bequignon, Karim Zaghbib, Gérard Royer, Audrey Colin, Giao Do-Pham, Christine Bodemer, Nicolas Ortonne, Annick Barbaud, Laurence Fardet, Olivier Chosidow, Pierre Wolkenstein, the French National Reference Center for Toxic Bullous Dermatoses, Marc Muraine, Pascal Joly, Florence Tetart, Olivier Dereure, Julie Waton, Brigitte Milpied, Anne-Marie Roguedas-Contios, Nadège Cordel, Claire Bernier, Maria-Polina Konstantinou

**Affiliations:** 1 Dermatology Department, AP-HP, Henri Mondor Hospital, 51 avenue du maréchal de Lattre de Tassigny, 94000 Créteil, France; 2 French National Reference Center for Toxic Bullous Dermatoses, Créteil, France; 3 Dermatology Department, Edouard Herriot Hospital, Lyon, France; 4 Dermatology Department, AP-HP, Necker Hospital, Paris, France; 5 Intensive Care Unit, AP-HP, Henri Mondor Hospital, Créteil, France; 6 Pharmacovigilance Department, AP-HP, La Pitié Salpêtrière Hospital, Paris, France; 7 Ophthalmology Department, Charles Nicolle Hospital, Rouen, France; 8 Ear Nose and Throat Department, AP-HP, Henri Mondor Hospital, Créteil, France; 9 Psychiatry Department, AP-HP, Henri Mondor Hospital, Créteil, France; 10 Ophthalmology Department, AP-HP, Henri Mondor Hospital, Créteil, France; 11 Dermatology Department, Centre Hospitalier Intercommunal de Créteil, Créteil, France; 12 Pathology Department, AP-HP, Henri Mondor Hospital, Créteil, France; 13 Dermatology Department, AP-HP, Tenon Hospital, Paris, France; 14 EA7379 EpiDermE (Epidemiologie en Dermatologie et Evaluation des Thérapeutiques), Université Paris-Est Créteil Val de Marne (UPEC), Créteil, France; 15 Université Paris-Est Créteil Val de Marne (UPEC), Créteil, France

**Keywords:** Stevens-Johnson syndrome, Lyell syndrome, Toxic epidermal necrolysis, Management, Treatment, Intensive care, Drug reaction, Causality

## Abstract

Epidermal necrolysis (EN) encompasses Stevens-Johnson syndrome (SJS, < 10% of the skin affected), Lyell syndrome (toxic epidermal necrolysis, TEN, with ≥30% of the skin affected) and an overlap syndrome (10 to 29% of the skin affected). These rare diseases are caused, in 85% of cases, by pharmacological treatments, with symptoms occurring 4 to 28 days after treatment initiation. Mortality is 20 to 25% during the acute phase, and almost all patients display disabling sequelae (mostly ocular impairment and psychological distress).

The objective of this French national diagnosis and care protocol (*protocole national de diagnostic et de soins*; PNDS), based on a critical literature review and on a multidisciplinary expert consensus, is to provide health professionals with an explanation of the optimal management and care of patients with EN. This PNDS, written by the French National Reference Center for Toxic Bullous Dermatoses was updated in 2017 (https://www.has-sante.fr/portail/jcms/c_1012735/fr/necrolyse-epidermique-syndromes-de-stevens-johnson-et-de-lyell). The cornerstone of the management of these patients during the acute phase is an immediate withdrawal of the responsible drug, patient management in a dermatology department, intensive care or burn units used to dealing with this disease, supportive care and close monitoring, the prevention and treatment of infections, and a multidisciplinary approach to sequelae. Based on published data, it is not currently possible to recommend any specific immunomodulatory treatment. Only the culprit drug and chemically similar molecules must be lifelong contraindicated.

## Background

Epidermal necrolysis (EN) encompasses Stevens-Johnson syndrome (SJS, < 10% of the skin affected), Lyell syndrome (also known as toxic epidermal necrolysis, TEN, with ≥ 30% of the skin affected) and an overlap syndrome (10 to 29% of the skin affected) [1]. This disease is extremely rare (incidence of two cases per million inhabitants per year), but particularly serious, with a mortality of 20 to 25% during the acute phase, and from 30 to 35% at 1 year [2].

Almost all patients display disabling sequelae. In 85% of cases [3], EN is caused by pharmacological treatments, with symptoms occurring 4 to 28 days after treatment initiation [1].

There are two phases in the course of the disease [4]:

1. The acute phase, which is particularly devastating and may be life-threatening, depending on the severity of cutaneous and mucosal lesions. Supportive care is the cornerstone of management. Mortality rates of 10 to 40% have been reported, depending on the percentage of the skin surface detached. Mortality increases by about 10% over the following weeks, due to the decompensation of other pre-existing chronic diseases [2];
2. The chronic phase, in which various sequelae occur in almost all patients (90% of patients at 1 year). The underlying mechanisms of these sequelae remain poorly understood. Physical sequelae mostly affect the skin (dryness, pigmentation, nail, hair and sweating abnormalities), eyes (dry-eye syndrome, cicatrizing conjunctivitis, foreshortening of the conjunctival fornices and symblepharon formation, corneal abnormalities, affecting visual function to various degrees), mouth (dryness, dental alterations, growth abnormalities affecting the permanent teeth in children), the genital organs and, more rarely, the digestive tract and bronchi. Psychological sequelae are frequent (post-traumatic stress disorder) [5].

The objective of this French national diagnosis and care protocol (*protocole national de diagnostic et de soins*; PNDS) is to provide health professionals with an explanation of the optimal management and care of patients with EN. Indeed, in 2005, the French health authorities recommended the formulation of a structured PNDS for rare skin diseases, to improve and harmonize their management nationwide and to facilitate the reimbursement of patients by social security. PNDS are developed through a critical literature review and a multidisciplinary expert consensus (www.has-sante.fr). However, due to the rarity of strong data-based evidence and systematic reviews in the field of rare skin diseases, the French health authorities preferred to make use of expert consensus for the development of these recommendations.

This PNDS, written by the French National Reference Center for Toxic Bullous Dermatoses in 2010 and updated in 2017 (https://www.has-sante.fr/portail/jcms/c_1012735/fr/necrolyse-epidermique-syndromes-de-stevens-johnson-et-de-lyell), can be used as a reference, by the family doctor, acting in concertation with a specialist, particularly during the joint establishment of the care protocol with the medical consultant and the patient for charge exemption.

However, the PNDS cannot cover all possible specific cases, comorbid conditions, therapeutic particularities or hospital care protocols. It cannot claim to cover exhaustively all the types of possible management or to replace the individual responsibility of doctors to their patients. Nevertheless, this protocol reflects the basic structure of management for patients with toxic bullous dermatoses in France, principally in hospital dermatology, burns or intensive care units.

## Diagnosis and initial evaluation

### Objectives

- ► To facilitate early diagnosis;
- ► To organize rapid transfer to a specialized unit;
- ► To provide patients and their families with information.

### Professionals involved and means required

The first professionals involved in patient care may be the family doctor or a specialist, particularly those prescribing “high-risk” drugs, but emergency doctors and hospital dermatologists are often rapidly implicated in primary or secondary care.

Emergency doctors, nurses, and hospital dermatologists play a key role in the process of diagnosis. These two groups of specialists (emergency doctors and dermatologists) should be preferentially targeted in training actions for doctors.

**Fig. 1:**
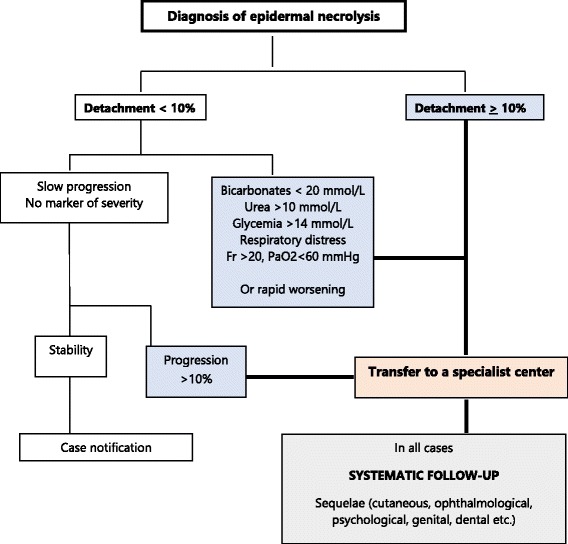
Algorithm for transfer to a specialist environment

Early recognition and withdrawal of the suspect drug are essential, to improve prognosis [6]. Transfer to a highly specialized center is a priority (dermatology department of the national Reference Center, burns or intensive care units), as soon as the diagnosis and seriousness of the patient’s condition have been confirmed (see the transfer algorithm in Fig. 1).

The diagnosis and initial evaluation of the patient in the acute phase require multidisciplinary cooperation, within a highly specialized center, in collaboration with the Reference Center, potentially involving: dermatologists; intensive care specialists; plastic surgeons from burn intensive care units; pediatricians; pulmonologists; specialists in infectious diseases; ophthalmologists; ENT specialists; stomatologists; gynecologists; urologists; gastroenterologists; psychiatrists, psychologists; clinical pharmacologists (involved in determining drug causality); dieticians; nurses and social workers.

The specialist team to which the patient is referred must have access to a nearby intensive care structure, together with the following resources: medical doctors and nurses trained in the management of patients with this condition, with appropriate beds; a warm environment; the possibility of applying and removing complex dressings under analgesia or anesthesia, if required; and a bacteriology laboratory. Supportive care is the cornerstone of the management of patients with EN, as shown by the lower mortality rates recorded in specialized units, such as the Reference Center [7].

### Initial diagnosis

#### Clinical diagnosis

Diagnosis is essentially clinical. Global and close-up digital photographs are useful for remote validation of the diagnosis. In cases of an otherwise apparently banal skin rash seen soon after onset, the alarm signs are: intense pain, high fever, mucosal involvement at several sites, purpuric macules, vesicles and cutaneous bullae, Nikolsky’s sign [1].

Clinical criteria for diagnosis:

- ► Purpuric macules or atypical targets, disseminated and not predominantly at the extremities;
- ► Vesicles, bullae;
- ► Epidermal detachment (“wet laundry” appearance);
- ► Nikolsky’s sign (detachment of the epidermis under finger pressure);
- ► Multifocal mucosal erosions (enanthem, bullae, erosions affecting the buccal cavity, nasopharynx, oropharynx, nose, eyes or genital/anal area);

The presence of at least three of these clinical criteria renders the diagnosis of EN probable.

Diagnosis cannot be established on the basis of laboratory tests or imaging.

Most of the biological and imaging tests required are those imposed by a situation of cutaneous or multiple organ failure.

#### Pathology examination

A skin biopsy, with pathology examination and direct immunofluorescence analysis, is required to confirm the diagnosis and to rule out other bullous diseases with a similar clinical presentation (such as linear IgA bullous dermatosis) [8].

Suggestive skin histology findings (i.e., nests of apoptotic keratinocytes with subsequent necrosis of the entire thickness of the epidermis and a dermal infiltrate consisting predominantly of lymphocytes) are required for diagnosis confirmation [9].

### Evaluation of causality

It is essential to note the precise chronology of the successive events leading to the disease and the chronology of drug intake during the months preceding hospitalization, comparing information from all possible sources (patient, family, family doctor, pharmacy etc.). The constitution of a drug intake timeline is desirable. EN typically occurs between four and 28 days after the introduction of the culprit drug [10]. Imputability analysis should take into account the half-lives of the drugs considered [11]. The suspect drug(s) should be withdrawn promptly, as this has been shown to improve prognosis [6].

In cases of doubtful causality, the Reference Center should be contacted urgently. The ALDEN imputability algorithm may facilitate the identification of the culprit drug(s) [3].

No drug origin is identified for 15% of EN cases [3]. Other examinations are therefore required to determine the etiology in these cases:

- ► Serological testing for *Mycoplasma pneumoniae* IgG and IgM (early testing and repeat testing after 3 weeks) and *Mycoplasma pneumoniae* PCR on throat swabs [12].
- ► Serological testing for other bacteria involved in atypical pulmonary infections (e.g. *Chlamydia* spp.), depending on the patient’s medical history [13].

Other examinations are also performed to evaluate the patient’s medical background:

- ► Serological tests for HIV;
- ► Tests for antinuclear antibodies (testing for underlying lupus), soluble nuclear antigens (SSA/SSB) [14].

### Differential diagnosis

- ► Other toxic bullous dermatoses: generalized bullous fixed drug reaction (lesions in well-delimited patches, little or no mucosal involvement, short induction time, notion of recurrence); drug-induced linear IgA bullous dermatosis (systematic direct immunofluorescence) [8, 15].
- ► Other severe cutaneous adverse reactions to drugs: acute generalized exanthematous pustulosis (confluent pustules progressing to fine superficial peeling, little or no mucosal involvement, histology) [16]; DRESS (drug reaction with eosinophilia and systemic symptoms [17]; typically, no detachment of the skin or mucosal involvement, but overlap syndromes may occur) [18].
- ► Autoimmune bullous dermatoses: idiopathic linear IgA dermatosis, pemphigus vulgaris, paraneoplastic pemphigus (histology and direct immunofluorescence, serum antibodies),
- ► Erythema multiforme major (typical or atypical targets, mucous membrane involvement very similar to that in EN), particularly in children and young adults. However, intermediate and difficult-to-classify presentations have been described [19], particularly in cases triggered by *Mycoplasma pneumoniae,* which some authors might classify as another conceptual entity (*Mycoplasma pneumoniae*-induced rash and mucositis) [20],
- ► Lupus with a Lyell-type presentation (signs associated with lupus, circulating antibodies) [14], dermatomyositis (DM) of the DM-Lyell type,
- ► Staphylococcal epidermolysis (SSSS, staphylococcal scalded-skin syndrome, very superficial peeling, markedly around the orifices, absence of mucosal erosion, histological features, underlying infection site) [4];
- ► Thermal or caustic burns, caustic dermatitis (medical history, absence of mucosal erosion, arrangement of skin lesions, histology).

### Classification and severity assessment

#### Classification

The classification of EN depends on the maximum affected area of the body (detached/detachable area) [1]:

- ► < 10%: Stevens-Johnson syndrome (SJS);
- ► 10 to 29%: overlap syndrome;
- ► ≥ 30%: Lyell syndrome or toxic epidermal necrolysis (TEN).

#### Prognosis

Death at the acute phase occurs in about 25% of cases (10 to 40%, depending on the percentage of the skin affected). The principal cause of death is sepsis and/or multiorgan failure (due to specific pulmonary or infectious causes in particular) [21–23].

The SCORTEN score is used, at admission, to evaluate the risk of death on the basis of seven clinical and biological parameters (Table 1) [24, 25].

**Table 1:** SCORTEN score (on admission)

Parameter	Value for SCORTEN (1 point)
Age	> 40 years
Cancer, hemopathy	yes
Percentage of skin detachment	> 10%
Pulse rate	> 120/min
Bicarbonates	< 20 mmol/L
Urea	> 10 mmol/L
Glycemia	> 14 mmol/L
Total score	Estimated risk of death in the acute phase
0–1	3%
2	12%
3	35%
4	58%
> 5	90%

#### Diagnosis communication

It is important to provide the patient and his/her family with information about the disease and its severity, upon arrival at the specialized unit. Patient confidentiality with respect to relatives should be respected in situations in which the drug inducing the condition is essentially specific for a particular disease (antiretroviral drugs, for instance).

A leaflet on the disease, containing the contact details of the patients’ association, should be given to the patients and their families, during the patient’s stay or at discharge, depending on the stress levels of the patient and his/her family, and psychological support should be proposed.

## Management of the acute stage

Treatment is essentially supportive, as no effective etiological treatment has been identified [4, 26, 27].

### Objectives

- ► To decrease mortality and morbidity by optimizing management;
- ► To prevent and limit long-term sequelae;
- ► To identify the drug or drugs likely to have caused the disease.

### Professionals involved

Therapeutic management of the patient during the acute phase is based on multidisciplinary cooperation coordinated by the specialist at the highly specialized center, in collaboration with the Reference Center. The multidisciplinary team involves the following specialists: dermatologist; intensive care specialist, or a plastic surgeon if the patient is admitted to the intensive care unit of a serious burns department; pediatrician; pulmonologist; infectious disease specialist; ophthalmologist; ENT specialist; stomatologist; gynecologist; urologist; gastroenterologist; psychiatrist, psychologist; dietician; nurses with specific training in the care of patients with this disease; social workers.

### Drug management

All suspect drugs should be stopped as soon as possible [6], but the continuity of management should be ensured. Numerous drugs have already been implicated in EN, but fewer than 10 products are responsible for half the cases occurring in Europe.

These most risky drugs are [10]:

- ► Allopurinol;
- ► Sulfonamide antibiotics (including sulfasalazine);
- ► Nevirapine;
- ► Antiepileptic drugs from the aromatic amine family: carbamazepine, oxcarbazepine, phenobarbital, phenytoin;
- ► Lamotrigine;
- ► Non-steroidal anti-inflammatory drugs (NSAIDs) of the oxicam family; Drugs associated with significant, but lower risk include [10]:
- ► Pantoprazole;
- ► Acetic acid NSAIDs;
- ► Various antibiotics, such as macrolides, quinolones, aminopenicillins, cephalosporins, and tetracyclines

The complete drug notoriety list is available from the Regiscar website (http://www.regiscar.org/Office_1.html).

Essential treatments not suspected to be responsible for the disease should not be stopped (the maintenance of such treatments during patient management in the acute phase prevents reticence concerning their subsequent use).

Drugs for which the risk of triggering EN is considered to be high can be used if indispensable and not suspected to be responsible for the disease in the patient treated.

In case of doubtful causality, the pharmacovigilance center, or the Reference Center should be contacted as soon as possible.

### Prevention of infections

To minimize the risk for nosocomial infections, asepsis rules must be rigorously respected. Hand hygiene and other infection control measures should be strictly applied. Strict rules should be defined for invasive procedures [26–28].

The high risk of infection justifies the repeated collection of skin specimens for bacteriological analyses (skin swabs or skin cultures on dedicated agar plates every 48 to 72 h), together with urine, blood and catheter swabs [29].

Antiseptics (antiseptic baths or aqueous chlorhexidine sprays, with rinsing) are used daily on the skin lesions.

Prophylactic antibiotic treatment is not recommended [21].

### Acute-phase supportive care

#### Fluid resuscitation and prevention of hypothermia

The supply of intravenous fluids and electrolytes should be adapted to the patient’s needs, on a case-by-case basis. Patients with a larger detached body surface area (i.e., ≥ 30%) will typically require larger daily fluid intakes. Peripheral catheters should be preferred for the vascular approach, with preferential implantation in uninjured skin regions. When required, intravenous and arterial lines should be inserted in uninjured skin regions, if possible. Catheters should be fixed with non-adhesive dressings. Catheters impregnated with an antiseptic (e.g., silver-sulfadiazine, chlorhexidine), which have been recommended for patients at high risk of catheter-related bacteremia, may be considered, except, of course, in cases of sulfonamide-induced EN [30].

The amount of fluid required for fluid resuscitation during the first 24 h can be estimated with numerous resuscitation formulas [31], none of which is optimal. We propose using the modified Brooke formula [32], as EN patients tend to display less extensive fluid loss than burns patients:

- Fluid volume (first 24 h) = 1.5 mL x % detached/detachable skin area x kg body weight
- The fluid volume should subsequently be adapted, essentially according to diuresis (objective: 0.5 to 1 mL/kg/h) and the extent of skin detachment in terms of body surface area. In case of shock and/or acute renal failure, hemodynamic monitoring is required, for the adjustment of fluid requirements and optimization of cardiovascular status. No specific monitoring tool has been recommended for the EN setting. However, non-invasive hemodynamic monitoring tools (e.g., Doppler echocardiography) should be considered, to limit the risk of catheter-related infections.

Room temperature must be kept between 28 and 32 °C (thermoneutral environment), to limit caloric losses and prevent hypothermia [33]. The use of warmed inspired gases and warming blankets should be considered.

#### Nutritional support

Early nutritional support by continuous enteral nutrition is recommended, with a target of 20–25 kcal/kg/day during the acute phase of illness (i.e.*,* the first week) increasing to 25–30 kcal/kg/day after the first week of management [34]. Routine gastric residual volume monitoring is not recommended.

Blood glucose levels should be monitored according to current guidelines [35]: intravenous insulin treatment should be initiated if two consecutive blood glucose determinations exceed 180 mg/dL (10 mmol/L) and should target an upper limit ≤180 mg/dL (10 mmol/L). Blood glucose concentrations should be monitored every 1–2 h until glucose values and insulin infusion rates are stable, and every 4 h thereafter.

#### Pain and psychological distress management

The evaluation and treatment of pain is a priority in acute phase management, particularly during wound care, which is performed several times daily. Pain should be assessed with appropriate tools, in sedated and non-sedated patients. All efforts should be made to provide the patient with the most comfortable environment possible. Patients and their families should be provided with music, radio or television and allowed to bring in some personal belongings to help reduce the stress and provide comfort. Opioids are required in most cases and their efficacy should be assessed with dedicated tools (e.g., a visual analog scale, VAS). Morphine is required if VAS score remains ≥4/10 [36]. General anesthesia may be necessary to achieve pain control. Alternatives to opioids include ketamine infusion during wound care for patients managed in the intensive care unit.

Active prevention of post-traumatic distress syndrome must be considered.

#### Management of acute respiratory failure

EN patients are at high risk of developing acute respiratory failure due to specific upper or lower (i.e., tracheobronchial) airway involvement or non-specific pulmonary complications, including pulmonary edema, pneumonia and atelectasis. Patients should, therefore, be monitored closely during the acute phase and transferred to the intensive care unit in case of respiratory deterioration. Chest X-ray and arterial blood gases should be obtained upon admission, for respiratory function assessment. EN-associated tracheobronchial lesions should be suspected when one of the following signs is present: productive cough (mucopurulent or bloody sputum), dyspnea, hypoxemia or radiological abnormalities. A bronchoscopy may be considered for diagnostic or therapeutic purposes, depending on the benefit/risk ratio [37].

Tracheal intubation and mechanical ventilation are necessary in about 25% of cases. Non-invasive ventilation is contraindicated because of skin lesions and the risk of upper airway obstruction due to laryngeal involvement. The need for tracheal intubation and mechanical ventilation must be anticipated. It is discussed, in practice, in cases of disturbed consciousness, hemodynamic instability, or acute respiratory distress, generally of multifactorial origin. Orotracheal intubation is often difficult and must be performed in an appropriate environment [22].

### Local skin care

The use of an appropriate bed (“air-fluidized” or equivalent) is recommended, to limit the deleterious effect of pressure on injured skin.

The removal of the detached epidermis is not recommended [38]. After disinfection with antiseptics and rinsing, erosions should be covered with non-sticky dressings (e.g. hydrocellular dressings) or white petroleum jelly. The types of local care practiced are highly diverse, and it is not possible to propose a single approach based on published data [26–28]. Some authors have proposed early wound coverage with synthetic skin substitutes, to reduce pain and accelerate epithelialization [39].

### Ophthalmological surveillance

Ophthalmological consultation should take place as soon as possible (within 24 h), to adapt treatment to the symptoms and to determine the frequency of surveillance. Symptomatic treatment aims to protect the cornea and to maintain hydration of the eye surface, to minimize subsequent problems, such as symblepharon [40, 41].

Supportive care is the mainstay of therapy. Local care should be administered every 2 hours and should involve the instillation of lubricant eye drops without preservatives and/or a vitamin A ophthalmic ointment. If necessary, symblepharon lysis will be performed regularly by the ophthalmologist, and by the application of a retropalpebral vitamin A-based ophthalmic ointment.

Antiseptic eye drops without preservatives should be used, if necessary. The use of topical corticosteroid therapy remains a matter of debate. Amniotic membrane transplantation may be considered during the acute phase, in the most severe cases [42].

### Surveillance of other mucosae

A rigorous clinical evaluation of the mucosal lesions is crucial during the acute phase and should be performed daily for accessible sites, including the outer ear. A specialized examination should be performed once weekly during the acute phase, for ENT and genital mucosae [43]. Antiseptic and analgesic mouth washes [44] should be used several times daily. The formation of genital adhesions can be prevented by daily foreskin mobilization, with the application of white petroleum jelly on the glans in men and of vaginal molds coated with white petroleum jelly in women [45].

### Specific immunomodulatory treatments

Based on published data, it is not currently possible to recommend any specific immunomodulatory treatment (e.g., steroids, intravenous immunoglobulins, cyclosporine or other immunosuppressants) for EN [28, 46–48].

Intravenous immunoglobulins have not improved mortality, even at high doses [49].

Prior exposure to steroids has been shown to increase the duration of healing, but with no beneficial impact on mortality [50]. High doses of methylprednisolone during the acute phase have no significant impact on mortality [46].

In an open monocenter trial on 29 patients conducted at our Reference Center, 3 mg cyclosporine/kg/day resulted in an absence of observed death, whereas 2.75 deaths were predicted by SCORTEN score, with control of epidermal detachment progression in 62% of patients [51]. Other retrospective studies have been performed, albeit with small samples. A recent Spanish study compared 26 patients treated with cyclosporine in one burns unit with 16 patients not treated with this drug in another burns unit in the same city. The authors then pooled their results with those of five previous case series. They found that cyclosporine decreases mortality by 60% [52, 53]. By contrast, in a recent single-center retrospective study conducted at our Reference Center, in which a propensity score method was used to match patients receiving cyclosporine plus supportive care with those who received supportive care only, we were unable to confirm these results [54]. However, this may reflect the low mortality rates for EN at our referral center (< 10%), due to optimized supportive care procedures, regardless of whether the patient is treated with cyclosporine or not. Consequently, the debate about the therapeutic potential of cyclosporine remains unresolved [48]. The main treatment remains optimized supportive care.

Thalidomide is contraindicated, due to its association with excess mortality, as shown in a prospective trial that was stopped early [55].

### Informing the patient and the family doctor

Upon discharge from hospital, patients should receive personalized information about the suspected or proven cause of the disease, the risk of sequelae, the need for follow-up and the possibilities for subsequent drug treatment.

On discharge, the patient should receive a written document listing the contraindicated and authorized drugs (allergy card and exhaustive list of prohibited medication), together with the contact details for the patients’ association, and the Reference Center.

The family doctor should be informed that the frequency, severity and progressive nature of the sequelae of EN require systematic follow-up, the costs and duration of which justify a request for 100% medical insurance coverage (Table 2).

**Table 2:** Summary for general practitioners

Epidermal necrolysis (EN) encompasses Stevens-Johnson syndrome (SJS, < 10% of the skin area affected), Lyell syndrome (also known as toxic epidermal necrolysis, TEN, with ≥30% of the skin affected) and an overlap syndrome (10 to 29% of the skin affected). EN is a very serious acute dermatological disease, mostly caused by pharmacological treatments and characterized by a sudden destruction of the epidermis and mucosal epithelia. The list of drugs implicated in this condition is very long, but fewer than 10 products are responsible for half the cases reported in Europe. These high-risk drugs are allopurinol, sulfonamide antibiotics (including sulfasalazine), nevirapine, antiepileptic drugs of the aromatic amine class (carbamazepine, oxcarbazepine, phenobarbital, phenytoin), lamotrigine, and non-steroidal anti-inflammatory drugs of the oxicam family. EN is very rare (about two cases per million inhabitants per year) and is a life-threatening emergency. Patients are usually not referred to specialist hospital departments until a mean of three days after the onset of symptoms, often due to late diagnosis.
When should a diagnosis of EN be suspected? What should be done? ∙ ∙ In cases of extensive skin rash and/or mucosal erosion ► With major changes in general state (hyperthermia, with body temperature > 39 °C and asthenia); ► On clinical examination: - Skin lesions: purpuric macules or atypical targets, vesicles and/or bullae, detachment of the skin spontaneously and on rubbing (“wet laundry” effect, Nikolsky’s sign), initially affecting the trunk, the proximal parts of the limbs and/or the face - Mucosal lesions: enanthem, bullae, erosions, affecting one or several mucosae ► Rapid progression of the symptoms over a period of seven to 10 days The association of these criteria should lead to a suspicion of EN. The general practitioner should immediately stop the drug suspected to be responsible and contact the Reference Center for Toxic Bullous Dermatoses as a matter of urgency. A transfer algorithm is provided in Fig. 1. Almost all patients suffer from sequelae, which may develop insidiously during the weeks or months following an apparently complete resolution of the condition. The most frequent sequelae are: ocular lesions (from dryness to symblepharons), post-traumatic stress disorder, skin pigmentation abnormalities, nail or hair disorders, genital, or dental lesions. Ocular sequalae are the type of lesions being potentially the most serious. For this reason, regular follow-up visits at the Referral Center are required. Only the molecules adjudged responsible for the patient’s condition and chemically similar molecules are contraindicated in the patient, and, as a precaution, in first-degree relatives. There is no justification for a contraindication of all drugs as a matter of principle or of other drugs reputed to be capable of inducing similar reactions but belonging to different chemical families from the drug implicated in the patient’s condition.
Consequently, the general practitioner should: ►Refer the patient to a specialized unit; ►Ensure that screening for sequelae is performed, in coordination with the Reference Center; ►Be careful not to prescribe either the drugs responsible for the condition or other chemically similar drugs to the patient in the future; ► If appropriate, constitute a dossier for social management or compensation, depending on the sequelae; ► Ensure that the patient receives psychological support.

## Follow-up

The follow-up period begins at the end of the acute phase and should be organized by the Reference Center, together with the family doctor. Its frequency depends on the nature and severity of the sequelae observed.

The follow-up should include two steps [4, 5, 56]:

- ► Systemic screening for sequelae.
- ► Management of sequelae by experienced specialists, in association with the family doctor and the social worker.

### Objectives

- ► To screen early for any EN sequelae, particularly those affecting the eyes, to facilitate the implementation of therapeutic measures and minimize their progression;
- ► To provide patients with all the help necessary to ensure as good a quality of life as possible;
- ► To help guide future medication use;
- ► To provide patients with information about advances in knowledge;
- ► To provide support for applications for compensation under the law relating to therapeutic contingencies (Law no. 2002–303 dated March 4, 2002 relating to the rights of patients and the quality of the health system) [57].

### Professionals involved

The key actors for the follow-up and screening of sequelae are the family doctor and the dermatologist from the Reference Center.

The hospital social worker provides assistance with administrative procedures, liaising with the administrative bodies and the social services of the sector, and with follow-up for integration at school, professional orientation, and providing information about the legislation relating to disability, in collaboration with occupational therapists, school doctors and handicap specialists, especially in case of severe visual impairment.

Psychological follow-up should be offered to all patients and, when required, to their families.

### Frequency of consultations at the reference center

Follow-up should be regular during the first year: usually, 2 months after the acute phase and then at 6 months and 1 year. The interval between visits may be lengthened or shortened, according to the sequelae observed. Further systematic follow-up is probably no longer justified if there are no sequelae at 1 year. Nevertheless, the patient and his/her family doctor should be given the contact details of a specialist center in case any problem occurs.

### Content of follow-up visits

Follow-up should include two steps.

#### Screening for sequelae

- ► Dermatological examination of the skin, hair and nails [58];
- ► Systematic ophthalmological examination, including checks for dry-eye syndrome and symblepharons, and a slit-lamp examination to guide subsequent surveillance and treatment;
- ► Buccal examination, including a dental examination, checks for dryness syndrome and a panoramic dental X ray in children [59];
- ► Gynecological examination and checks for synechia and vulvovaginal dryness [60];
- ► ENT examination [43];
- ► Clinical pulmonary examination and respiratory function testing, preferably 2 months after the acute phase and at 1 year if the patient has symptoms;
- ► Psychiatric evaluation and offer of psychological support to the patient and his/her family (due to the high frequency of post-traumatic stress syndrome in these patients) [61].

#### Management of sequelae

The management of sequelae is guided by patient interview and the results of the clinical evaluation. Sequelae are managed by experienced specialists, liaising with the family doctor and the Reference Center, according to the symptoms. The specialists involved may be: ophthalmologists specializing in the eye surface; gynecologists or urologists; stomatologists/dentists (prevention and correction of dental problems); psychiatrists and psychologists; pulmonologists (pulmonary function tests, pulmonary imaging); gastroenterologists (endoscopy); plastic surgeons (reconstruction surgery); dermatologists skilled in the use of reconstruction techniques (lasers etc.); other specialists.

#### Management of ophthalmological complications

For serious sequelae, the patient is managed by a team of specialists in diseases of the surface of the eye.

The use of gas-permeable scleral lenses has been shown to have beneficial effects on pain and photophobia, with significant improvements in vision and quality of life [62].

Other types of treatment may be useful and should be discussed with an ophthalmological service experienced in the management of this condition: cyclosporine eye drops [63], epilation in cases of trichiasis, by electrolysis or another technique, surgical fornix reconstruction with amniotic membrane transplantation or mucous membrane grafting [64], keratoprosthesis.

### Investigation of causality

This investigation involves pharmacovigilance and allergology (tests performed in vitro and in vivo, which should not be performed, for the patch tests, until at least three to 6 months after complete resolution of the acute phase) [65, 66]. It may take several weeks of investigation to come to a firm conclusion about the causal agent. The patient should then receive an allergy card listing all the molecules contraindicated.

The pharmacovigilance agencies should be systematically notified of all such drug-related adverse events [67].

### Therapeutic education: Drug treatments

Patients who have had EN need to be involved in their own therapeutic education: understanding of the disease, need for follow-up, subsequent use of medication and possible changes in lifestyle (depending on the sequelae present).

Only the molecules implicated in the disease and chemically similar molecules are contraindicated (to prevent possible cross-reactions). Whilst our understanding of the genetic factors predisposing individuals to this condition remains incomplete, it is prudent to extend this contraindication to all first-degree relatives of the patient [68]. There is no justification for the contraindication of all drugs reputed to cause EN.

## Conclusion

We report here for the international medical community the French guidelines for EN management. A prompt withdrawal of the culprit drug(s), the transfer of the patient to a specialized unit and maximal supportive care are the cornerstones of patient management.
